# Microinvasive carcinoma of the uterine cervix in a 14-year-old adolescent: case report and literature review

**DOI:** 10.1590/S1516-31802009000200010

**Published:** 2009-07-06

**Authors:** Carla Vitola Gonçalves, Silvana Maria Quintana, Alessandra Cristina Marcolin, Geraldo Duarte, Juvenal Soares Dias da Costa, Fabine Karam, Mônia Steigleder

**Affiliations:** 1 MD. Adjunct professor, Mother and Child Department, Fundação Universidade Federal do Rio Grande (FURG), Rio Grande do Sul, Brazil.; 2 MD. Assistant professor, Department of Gynecology and Obstetrics, Faculdade de Medicina de Ribeirão Preto (FMRP), Universidade de São Paulo (USP), Ribeirão Preto, São Paulo, Brazil.; 3 MD. Medical gynecologist and obstetrician, Hospital das Clínicas (HC), Faculdade de Medicina de Ribeirão Preto (FMRP), Universidade de São Paulo (USP), Ribeirão Preto, São Paulo, Brazil.; 4 MD, PhD. Titular professor, Department of Gynecology and Obstetrics, Faculdade de Medicina de Ribeirão Preto (FMRP), Universidade de São Paulo (USP), Ribeirão Preto, São Paulo, Brazil.; 5 MD. Adjunct professor, Department of Social Medicine, Universidade Federal de Pelotas (UFPel), Pelotas, Rio Grande do Sul, Brazil.; 6 MD. Medical resident in Gynecology and Obstetrics, Fundação Universidade Federal do Rio Grande (FURG), Rio Grande do Sul, Brazil.; 7 Medical student, Fundação Universidade Federal do Rio Grande (FURG), Rio Grande do Sul, Brazil.

**Keywords:** Uterine cervical neoplasms, Colposcopy, Papillomavirus infections, Adolescent, Uterine cervical diseases., Neoplasias do colo uterino, Colposcopia, Infecções por papilomavirus, Adolescente, Prevenção de câncer de colo uterino.

## Abstract

**CONTEXT:**

Cancer of the uterine cervix is rare during adolescence. The reported rates are 0/100,000 adolescents aged 10 to 19 years and 1.7/100,000 women aged 20 to 24 years. However, several studies have shown increasing incidence of preneoplastic lesions at increasingly early ages.

**CASE REPORT:**

This paper reports a case of microinvasive carcinoma of the uterine cervix in a 14-year-old patient with menarche at 10 years of age and first coitus at 12 years of age. The objective of the present report was to alert gynecologists and pediatricians regarding the need for cervical carcinoma prevention among sexually active adolescents, based on educational programs that explain the purpose of colpocytological examinations and encourage their use, along with condom use and limitation of the number of sexual partners.

## RESUMO

**CONTEXTO:**

O câncer do colo uterino é raro na adolescência, apresentando taxas de incidência de 0/100.000 adolescentes com idade entre 10 a 19 anos e de 1,7/100.000 mulheres com idade entre 20 e 24 anos. Entretanto, vários estudos mostram a incidência crescente das lesões pré-neoplásicas em idade cada vez menores.

**RELATO DE CASO:**

Este artigo relata um caso de carcinoma microinvasor do colo uterino em paciente de 14 anos com menarca aos 10 anos e a primeira relação aos 12 anos. Com este relato objetivamos alertar a comunidade médica, principalmente ginecologistas e pediatras, para a prevenção do carcinoma cervical entre adolescentes com vida sexual ativa. Além da realização de projetos educativos que divulguem a finalidade do exame citopatológico, o uso de preservativos e o controle do número de parceiros sexuais.

## INTRODUCTION

Cancer of the uterine cervix is the second most frequent neoplasia affecting women worldwide. The World Health Organization (WHO) has estimated that more than one million women are currently affected by this disease, and that approximately 95% of the cases occur in developing countries.^1^ In Brazil, cervical cancer is the third most common neoplasia among women. The coefficient of mortality due to cervical carcinoma in 2005 was 4.8 cases/100,000 women, representing the fourth largest cause of death due to cancer among women.^2^

The causative agent of cervical cancer is the human papillomavirus (HPV), which is mainly transmitted sexually and is more prevalent among women whose sexual initiation occurred before the age of 16-18 years and who have had multiple partners.^2^ The WHO has estimated that 25 to 30% of women younger than 25 years of age are infected with high-risk HPV.^1^

Several studies have shown increasing incidence of preneoplastic lesions at increasingly early ages.^3^^,^^4^ An increase in high and low-grade lesions of the cervix among young women who started their sexual activity during adolescence, with constantly changing partners, is also being observed in Brazil.^5^^,^^6^^,^^7^^,^^8^ However, cervical malignancy is rare during adolescence and estimates from the National Cancer Institute’s Surveillance, Epidemiology and End Results (SEER) program indicated cervical cancer rates of 0/100,000 adolescents aged 10 to 19 years and 1.7/100,000 women aged 20 to 24 years, from 1995 to 1999.^9^

## CASE REPORT

D.E.F.O., a 14-year-old nulliparous girl with menarche at 10 years of age and first coitus at 12 years of age sought a gynecological consultation for the first time, for counseling about contraception. She reported that she had had a single sexual partner since she began sexual activity two years earlier. She was not using any hormonal contraceptive and she reported sporadic use of condoms. General and specific physical examinations did not reveal any noteworthy features.

However, colpocytological examination (February 15, 2007) revealed a high-grade intraepithelial lesion. The patient underwent colposcopy on March 8, 2007, which revealed major abnormal findings and a cervical biopsy was obtained by means of a high-frequency loop electrosurgical excision procedure (LEEP). The anatomopathological examination on the specimen revealed the presence of a high-grade intraepithelial cervical squamous lesion (cervical intraepithelial neoplasia, CIN, grade III and *in situ* carcinoma).

The patient underwent conization by means of LEEP on March 28, 2007. Histopathological examination on the specimen revealed the presence of microinvasive epidermoid carcinoma infiltrating the cervical wall to a depth of 1 mm and extending horizontally 1 mm, with neoplasia running along the vascular bed of the stroma, multiple foci of *in situ* carcinoma and cell architectural changes due to HPV. It was decided to monitor the patient with colpocytological examinations at three-month intervals during the first postoperative year. The first post-conization evaluation was performed on June 28, 2007, and the colpocytological findings were considered to be satisfactory, with inflammatory changes.

## DISCUSSION

A systematic review of the literature was developed by searching in the PubMed, Cochrane Library, Excerpta Medica Database (Embase), Literatura Latino Americana e do Caribe em Ciências da Saúde (Lilacs), Scientific Electronic Library Online (SciELO) and ADOLEC databases. The search strategies and the results are shown in Table 1. Most of the references related to HPV infection and to the vaccine against this virus, applied to adolescents. In the few articles on the presence of colpocytological changes among adolescents, there were reports of high incidence of low-grade lesions and warnings about increasing frequency of findings of high-grade lesions in this group. Currently, the incidence of invasive cancer of the uterine cervix among adolescents up to 19 years of age is considered to be almost nonexistent, with no reports of this type of case.^3^^,^^4^^,^^5^^,^^6^^,^^7^^,^^8^ On this basis, the objective of the present case report was to alert the medical community, and gynecologists and pediatricians in particular, to the need for cervical cancer prevention practices among sexually active adolescents.

**Table 1.** Systematic review of the literature

Database	Search strategy	Results
PubMed	Uterine cervical neoplasms AND Colposcopy AND Papillomavirus infections AND Adolescent AND Cervical neoplasm prevention	32 articles 11 case-control studies 7 cross-sectional prevalence studies 6 review articles on screening 5 guidelines 2 clinical trials 1 case report
Cochrane	Uterine cervical neoplasms AND Adolescent	17 articles 11 clinical trials 4 systematic reviews 2 economic evaluations
Embase (Excerpta Medica Database)	Uterine cervical neoplasms AND Adolescent	8 articles 2 case-control studies 4 cross-sectional prevalence studies 1 guidelines 1 clinical trial
Lilacs (Literatura Latino-Americana e do Caribe em Ciências da Saúde)	Neoplasia do colo uterino AND Adolescente	4 cross-sectional prevalence studies
SciELO (Scientific Electronic Library Online)	Neoplasia do colo uterino AND Adolescente	1 cross-sectional prevalence study
ADOLESC ADOLEC	Neoplasia do colo uterino AND Adolescente	20 articles 9 cross-sectional prevalence studies 7 review articles on screening 4 case-control studies

MeSH = Medical Subject Headings; DeCS = Descritores em Ciências da Saúde.

The WHO, the Brazilian National Cancer Institute (Instituto Nacional do Câncer, INCA) and the Brazilian Health Ministry recommend that all currently or previously sexually active women, regardless of age, should undergo periodic preventive cervical cancer examinations. Nevertheless, even though the first mobilization for early detection of cervical cancer in Brazil occurred in 1998, this disease is still a public health problem in this country.^1^^,^^2^ The estimated coverage of the Papanicolaou test is still low.^2^

In the state of Acre, 301 new cases of cervical cancer were recorded in 2000, 116 of them in women younger than 30 years of age. In view of this observation, a study was conducted in that state in order to determine the frequency of precursor lesions for cervical carcinoma among women aged 15 to 29 years. The study revealed that 6.4% of women aged 15 to 29 years and 6.3% of women aged 20 to 29 years presented intraepithelial lesions, as determined by cytological examination. The study did not specify the grade of the lesion.^8^

The case reported here stands out because of the diagnosis of microinvasion from the lesion in such a young patient, who had been sexually active for only two years. A study conducted on 702 sexually active adolescents aged 12 to 19 years revealed that the prevalence of intraepithelial lesions was 8.4%. In that study, 3.0% presented high-grade lesions, and one case of carcinoma was detected. The authors pointed out that the mean interval between the beginning of sex life and the diagnosis of the lesions was 1.7 years, and that high-grade lesions occurred within the first two years of sexual activity in 57% of the infected patients.^9^

An analysis conducted by the Lower Genital Tract Pathology and Colposcopy Sector of the General Hospital of Nova Iguaçu, Brazil, on a group of women aged 13 to 24 years revealed that there was no significant difference in any grade of intraepithelial cervical lesion between the group of 13 to 19 years of age and the group of 20 to 24 years of age. In that study, two cases of microinvasive carcinoma were detected, one in each group, and three cases of invasive carcinoma were detected in the 20 to 24-year group.^10^

Some authors have reported that adolescent girls are more susceptible to HPV infection and that precursor lesions for cervical carcinoma progress more rapidly in this group. These findings may be related to systemic and local (lower genital tract) immunological immaturity, to greater prevalence of exposure of the cylindrical epithelium in the ectocervix with consequent metaplasia, and to low incidence of the use of barrier methods.^2^^,^^11^

Finally, we would like to emphasize that, although the natural history of cancer of the cervix is characterized by slow evolution, precursor lesions are being detected with increasing frequency among adolescents, where they seem to progress more rapidly. Since the prognosis for cervical carcinoma depends on the extent of the disease at the time of diagnosis, it is extremely important to develop programs for cervical cancer prevention among younger women who are not targeted by official governmental programs.

## References

[1] World Health Organization (2006). Comprehensive cervical cancer control: A guide to essential practice.

[2] Brasil. Ministério da Saúde. Secretaria de Atenção à Saúde. Instituto Nacional de Câncer (2007). Coordenação de Prevenção e Vigilância de Câncer. Estimativa 2008: Incidência de câncer no Brasil.

[3] Moscicki AB (2005). Cervical cytology testing in teens. Curr Opin Obstet Gynecol.

[4] Moore K, Cofer A, Elliot L, Lanneau G, Walker J, Gold MA (2007). Adolescent cervical dysplasia: histologic evaluation, treatment, and outcomes. Am J Obstet Gynecol.

[5] Utagawa ML, Pereira SM, Cavaliere MJ, Maeda MY, Shih LW, Shirata NK (1998). Cervical intraepithelial neoplasia in adolescents: study of cytological findings between 1987 and 1995 in São Paulo State-Brazil. Arch Gynecol Obstet.

[6] Longatto A, Etlinger D, Gomes NS, Cruz SV, Cavalieri MJ (2003). Freqüência de esfregaços cérvico-vaginais anormais em adolescentes e adultas: revisão de 308.630 casos [Frequency of abnormal uterine cervix smears from adolescents and adult women: review of 308.630 cases]. Rev Inst Adolfo Lutz.

[7] Leal EAS, Leal OS, Guimarães MH, Vitoriano MN, Nascimento TL, Costa OLN (2003). Lesões precursoras do câncer de colo em mulheres adolescentes e adultas jovens do município de Rio Branco - Acre [Cervical cancer precursor lesions in adolescent and young adult women of Rio Branco - Acre]. Rev Bras Ginecol Obstet.

[8] Monteiro DLM, Trajano AJB, Silva KS, Russomano FB (2006). Doença cervical pré-invasiva e câncer cérvico-uterino em adolescentes brasileiras: prevalência e fatores associados [Pre-invasive cervical disease and uterine cervical cancer in Brazilian adolescents: prevalence and related factors]. Cad Saúde Pública = Rep Public Health.

[9] Insinga RP, Glass AG, Rush BB (2004). Diagnoses and outcomes in cervical cancer screening: a population-based study. Am J Obstet Gynecol.

[10] Nascimento MI, Pires ES, Gil DQ (2005). Características de um grupo de adolescentes com suspeita de neoplasia intra-epitelial cervical [Characteristics of a group of adolescents with suspected cervical intraepithelial neoplasia]. Rev Bras Ginecol Obstet.

[11] Kahn JA, Rosenthal SL, Succop PA, Ho GY, Burk RD (2002). The interval between menarche and age of first sexual intercourse as a risk factor for subsequent HPV infection in adolescent and young adult women. J Pediatr.

